# Advancing intraoperative magnetic tracing using 3D freehand magnetic particle imaging

**DOI:** 10.1007/s11548-021-02458-2

**Published:** 2021-07-31

**Authors:** Samaneh Azargoshasb, Lennert Molenaar, Giuseppe Rosiello, Tessa Buckle, Danny M. van Willigen, Melissa M. van de Loosdrecht, Mick M. Welling, Lejla Alic, Fijs W. B. van Leeuwen, Alexander Winter, Matthias N. van Oosterom

**Affiliations:** 1 Interventional Molecular Imaging Laboratory, Department of Radiology, Leiden University Medical Center, Leiden, The Netherlands; 2 Magnetic Detection & Imaging Group, Technical Medical Centre, University of Twente, Enschede, The Netherlands; 3 Department of Urology, Onze-Lieve-Vrouw Hospital, Aalst, Belgium; 4 ORSI Academy, Melle, Belgium; 5 Department of Urology and Division of Experimental Oncology, URI, Urological Research Institute, IRCCS San Raffaele Scientific Institute, Milan, Italy; 6 Department of Urology, Netherlands Cancer Institute – Antoni van Leeuwenhoek Hospital, Amsterdam, The Netherlands; 7 University Hospital for Urology, Klinikum Oldenburg, School of Medicine and Health Sciences, Carl Von Ossietzky University Oldenburg, Oldenburg, Germany

**Keywords:** Magnetic particle-guided surgery, Fluorescence-guided surgery, Augmented reality, Surgical navigation, Penile cancer

## Abstract

**Purpose:**

Sentinel lymph node biopsy is a routine procedure for nodal staging in penile cancer. Most commonly, this procedure is guided by radioactive tracers, providing various forms of preoperative and intraoperative guidance. This is further extended with fluorescence imaging using hybrid radioactive–fluorescence tracers. Alternatively, a magnetic-based approach has become available using superparamagnetic iron-oxide nanoparticles (SPIONs). This study investigates a novel freehand magnetic particle imaging and navigation modality (fhMPI) for intraoperative localization, along with a hybrid approach, combining magnetic and fluorescence guidance.

**Materials and methods:**

The fhMPI set-up was built with a surgical navigation device, optical tracking system and magnetometer probe. A dedicated reconstruction software based on a look-up-table method was used to reconstruct a superficial 3D volume of the SPION distribution in tissue. For fluorescence guidance, indocyanine green (ICG) was added to the SPIONs. The fhMPI modality was characterized in phantoms, ex vivo human skin and in vivo porcine surgery.

**Results:**

Phantom and human skin explants illustrated that the current fhMPI modality had a sensitivity of 2.2 × 10^–2^ mg/mL SPIONs, a resolving power of at least 7 mm and a depth penetration up to 1.5 cm. Evaluation during porcine surgery showed that fhMPI allowed for an augmented reality image overlay of the tracer distribution in tissue, as well as 3D virtual navigation. Besides, using the hybrid approach, fluorescence imaging provided a visual confirmation of localized nodes.

**Conclusion:**

fhMPI is feasible in vivo, providing 3D imaging and navigation for magnetic nanoparticles in the operating room, expanding the guidance possibilities during magnetic sentinel lymph node procedures. Furthermore, the integration of ICG provides the ability to visually refine and confirm correct localization. Further clinical evaluation should verify these findings in human patients as well.

## Introduction

In cancer therapy, reliable information about the regional lymph node (LN) status is of great importance for accurate staging and optimal planning of treatment. Despite advances in non-invasive imaging [e.g. positron emission tomography (PET)], LN dissection followed by histopathological examination continues to be the most accurate modality to identify (micro-) metastases [1]. Sentinel LN (SLN) surgery is based on the premise that cancer metastases pass through one gatekeeper LN (i.e. a SLN) or a group of SLNs before spreading further [2, 3]. Focusing histopathological examination on this subset of gatekeeper LNs, it becomes feasible to perform ultrastaging of the specimens (e.g. fine serial sectioning and immunohistochemistry analysis), increasing the change to find low-volume metastases. Sampling of these SLNs thus allows for staging of the lymphatic spread.

Advantages of such a targeted SLN biopsy include tailoring of the surgical procedure to the individual patient, allowing to distinguish between patients who need extensive nodal dissection from those who would not gain an oncological benefit from such an invasive dissection. This does not only increase the specificity of the approach, but also results in lower morbidity [4]. For these reasons, SLN biopsy has become routine for nodal staging in a.o. breast cancer, penile cancer and melanoma [5–7].

Different technologies are used for marking, preoperative visualization and intraoperative identification of SLNs. Most common and established is the use of radioactive tracers (e.g. ^99m^Technetium-nanocolloid), allowing for preoperative imaging [i.e. scintigraphy and single-photon emission computed tomography/computed tomography (SPECT/CT)], as well as intraoperative detection of the SLNs using gamma detection probes and mobile gamma cameras [8]. Using the input of such gamma probes and cameras, even freehand SPECT (fhSPECT) scans have been realized in the operating room. This particular technology allows for 3D images to be generated in the operating room, a concept that further facilitates accurate navigation towards the SLNs [9, 10]. Use of the hybrid tracer indocyanine green [ICG]-^99m^Tc-nanocolloid, a tracer that has proven its potential in a.o. melanoma and penile cancer [11, 12], has helped complement the above-mentioned radioguidance concepts with real-time fluorescence imaging [13–15]. In parallel to these efforts, the use of magnetic tracers [e.g. superparamagnetic iron-oxide nanoparticles (SPIONs)] has also demonstrated potential in SLN procedures (e.g. penile cancer, melanoma, breast cancer and prostate cancer [16–19]. Similar to the radioguidance approach, the SLNs can be visualized preoperatively with magnetic resonance imaging (MRI) [20, 21]. Intraoperatively, the SPIONs are detected by a handheld magnetometer probe [20, 21]. Similarly, as with a gamma probe in the radioguided approach, this magnetometer probe provides audible and numerical feedback with respect to the nodal tracer uptake. Unfortunately, to date, intraoperative magnetic tracing has been limited to the use of such a probe only.

To advance the intraoperative visualization of the SLNs in a magnetic approach, this study has investigated the use of a hybrid ICG-SPION approach and intraoperative freehand magnetic particle imaging (fhMPI). These technologies were evaluated in phantom measurements, ex vivo human skin explants and during SLN surgery in porcine models, focused towards applications in the groin, which is the primary lymphatic landing site for penile cancer and melanoma.

## Methods

### Imaging tracers

A commercially available SPION tracer, Magtrace (Endomagnetics Ltd, Cambridge, UK), was used, consisting of iron-based nanoparticles dissolved in saline. The nanoparticles contain multiple iron-oxide cores (single-core diameter: 3.5–10 nm), agglomerated and encapsuled by a carboxydextran coating, bringing the hydrodynamic diameter of the particles to 45–65 nm. To provide additional guidance during surgery and investigate a possible hybrid surgical workflow, the combined use of magnetics and fluorescence was utilized for all evaluations. To this end, 50 µL of the fluorescent dye ICG (5 mg/mL dissolved in water; Verdye, Diagnostic Green GmbH, Aschheim-Dornach, Germany) was added to a 2 mL vial of SPIONs (28 mg/mL).

Additionally, to investigate possible non-covalent complex formation between ICG and SPION, the ICG-SPION mixture was investigated with a size-exclusion column, as previously described [22, 23]. To this end, 2.5 μL ICG solution was added to 100 μL Magtrace solution. After shaking for 30 min at room temperature, unbound ICG molecules were removed by purifying the mixture over a PD10 size-exclusion column (GE-Healthcare, Chicago, USA), using saline as eluent. Both absorption and fluorescent emission spectra were recorded of the collected column fractions.

### Freehand magnetic particle imaging and navigation set-up

**Fig. 1:**
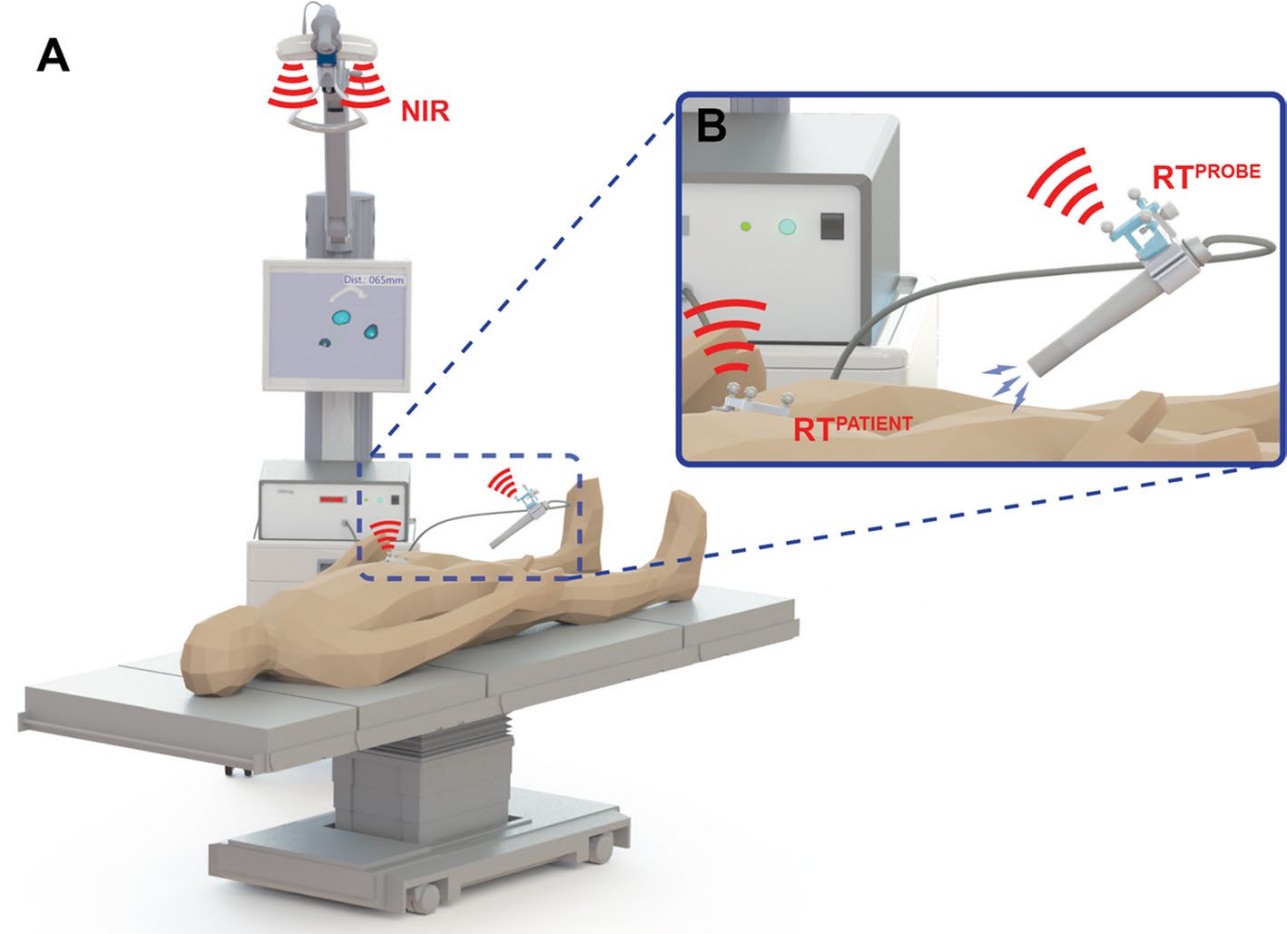
Schematic overview of the fhMPI imaging and navigation set-up. **a** Overview of the complete set-up, displaying the surgical navigation device, the handheld magnetometer probe and the near-infrared (NIR) optical tracking. **b** Magnification of the handheld magnetometer probe, displaying the probe reference target (RT^probe^) and the patient reference target (RT^patient^)

The main components of the fhMPI imaging and navigation set-up (Fig. 1) include:

- A DeclipseSPECT navigation system (SurgicEye GmbH, Munich, Germany) with an integrated near-infrared (NIR) optical tracking system (Polaris Vicra; Northern Digital Inc., Waterloo, Canada) [24];
- A 1D handheld magnetometer probe (DiffMag; MD&I group, University of Twente, The Netherlands [25]), based upon a differential magnetometry principle. The 22 mm diameter DiffMag probe consists of an excitation coil and a set of detection coils in a gradiometric configuration, assessing the difference between signal generated by SPIONs and background noise.
- An Arduino-based signal processing unit (Arduino Uno; Arduino AG, Italy) linking the output of the DiffMag probe to a custom version of the Declipse 6.0 software (SurgicEye GmbH).

To detect the position and orientation (3D pose) of the magnetometer probe in the operating room, a three-fiducial reference target (RT^probe^) was placed at the distal end of the probe and optically tracked (Fig. 1). This reference target consisted of a sterile part placed outside of the sterile probe cover, and a 3D printed plastic clip attached directly to the probe under the sterile cover, calibrated using previously described procedures [26]. To track the pose of the tissue of interest, a second, but geometrically different, three-fiducial reference target (RT^patient^) was placed close to the volume of interest on a (semi-)rigid part of the scanning objective (i.e. phantom, ex vivo tissue or porcine model). During the fhMPI application, a direct line-of-sight was maintained between the NIR optical tracking system and both the RT^patient^ and RT^probe^.

Using the custom Declipse software algorithm, a superficial 3D volume was reconstructed, representing the SPION distribution in tissue (i.e. the aforementioned fhMPI scan). This reconstruction was based on a look-up-table method, common for freehand imaging as described previously [14, 27]. The look-up-table was generated by measuring the DiffMag magnetometer probe response to a SPION point source (46 μg) in air over a volume of 31 × 31 × 9 mm at a resolution of 1 mm, using a robotic x–y–z arm (Meca500, Mecademic, Montreal, Canada), similar as reported previously for freehand fluorescence imaging [14].

The settings for the eventual reconstruction were: 3 × 3 × 3 mm voxel size, 3 mm Gaussian filter and a 180 × 180 × 120 mm volume of interest. During fhMPI scan acquisition, the magnetometer probe was manually moved over the tissue in the volume of interest, measuring at as many different positions and orientations as possible, keeping the total acquisition time below 5 min. Even though the current magnetometer probe employs differential magnetometry to correct for interference by nearby metal objects (e.g. surgical clamps) [25], some ‘noise counts’ are still observed when the probe is moved dynamically. To minimize noise in the fhMPI scans during acquisition, magnetometer measurements below 20 counts were excluded.

### Characterization of the freehand magnetic particle imaging and navigation modality

Feasibility of the fluorescent and magnetic particle tracking in human tissue was investigated using explanted human skin samples. To this end, 3 deposits of each 100 µL ICG-SPION tracer were injected subcutaneously. In this setting, each deposit roughly represented a typical SLN uptake value as found in prostate/penile cancer (i.e. 5% of the typically injected dose [23, 28]). FhMPI imaging and navigation were evaluated, as well as fluorescence imaging using a PDE-mod open surgery fluorescence camera (Hamamatsu Photonics k.k., Hamamatsu, Japan).

To investigate the sensitivity of the novel fhMPI modality, a well plate was used to create a dilution series of SPION tracer. Divided over 14 wells, each well was filled with 100 µL of ICG-SPION, ranging from 2.8 mg/mL down to 1.7 × 10^–4^ mg/mL using a human serum albumin solution (40 mg/mL in H_2_O). For each well, a background-corrected magnetometer signal was recorded and a fhMPI scan was made, evaluating the minimal tracer concentration for successful reconstruction. All measurements were taken in triplicate.

The resolving power of the fhMPI modality was assessed using a plastic plate phantom [14], containing 7 well pairs with each a decreasing distance between the edges (ranging from 18 to 4 mm). Each of the individual wells was filled with 100 µL ICG-SPION tracer. With the unobstructed liquid level present at the surface of the phantom (i.e. a depth of 0 cm), any depth penetration effects were avoided in this set-up. The fhMPI scan was acquired in triplicate for each well pair.

Depth penetration of fhMPI was evaluated using explanted human skin. 100 µL of ICG-SPION was inserted at different depths into the tissue (i.e. 0, 0.5, 1, 1.5 and 2 cm) and investigated using magnetometer counts and the novel fhMPI method.

### In vivo porcine evaluation

In vivo feasibility of the fhMPI set-up was investigated in porcine models (n = 2, four groins; weight per animal approximately 40 kg). ICG-SPION was administered in the operating room, using subcutaneous injections in both legs (0.5 mL each). Immediately after administration, the injection sites were massaged for ~ 15 min to promote lymphatic drainage. The time between injection and localization was 45 min. The RT^patient^ was placed on the skin surface at the pubic bone location. The localization started with transcutaneous magnetic tracing using the magnetometer probe, followed by fhMPI scan acquisition of the anatomy of interest and an additional check with fluorescence imaging (Firefly fluorescence endoscope and endoscope plus models, DaVinci Xi, Intuitive Inc., Sunnyvale CA, USA). The information of these modalities was used to guide the incision and localize individual SLNs. Upon exposure of the node, fluorescence imaging was performed again to confirm the nodal target. After surgical retrieval, the SLN specimens were also imaged ex vivo. Animal experiments were performed under approval of the local ethical committee.

### Pathology

To confirm the presence of SPIONs in the excised SLNs, the surgical specimens were fixed in 4% buffered formaldehyde for 1 week at room temperature and then processed for paraffin embedding. Next, sections of 4 μm were cut and dried overnight at 37 °C. Perl’s Prussian blue iron staining was used to investigate for SPION presence, while the nuclei were stained with nuclear fast red to provide anatomical context.

## Results

### Hybrid SPION tracer

**Fig. 2:**
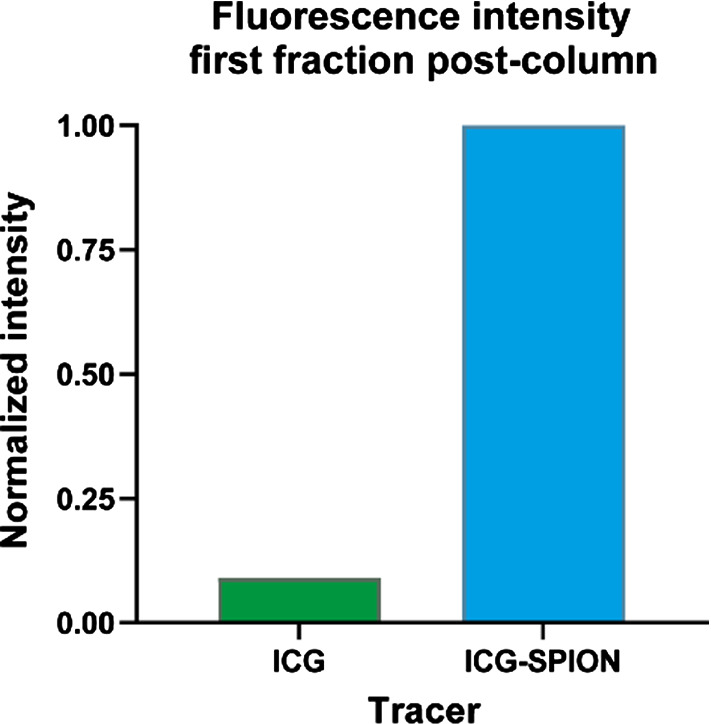
Visualization of the size-exclusion column measurements for ICG-SPION. Fluorescence intensity is shown of the first fraction after purification of a column with ICG and a column with ICG-SPION. This clearly shows a rise of fluorescence emission at 810 nm for ICG-SPION, indicating that at least part of the ICG has bound non-covalently to the SPION particles. The intensity values were corrected by subtracting the SPION background intensity and normalized to that of ICG-SPION

Similar as was shown for ICG-^99m^Tc-nanocolloid [22, 23], after purifying ICG-SPION over a size-exclusion column, ICG fluorescence should be minimally present in the first eluting column fraction, unless ICG molecules were directly bound to SPION particles. Figure 2 visualizes the fluorescence intensity of the first eluting fraction for an ICG column and an ICG-SPION column, clearly showing an increase in fluorescence measured at 810 nm. Analysing this fraction, we were able to identify the distinct fluorescence absorption and emission signatures of ICG in the fraction that contains SPIONs. This indicates that at least part of the ICG has bound non-covalently to the SPION nanoparticles,

### Characterization of the freehand magnetic particle imaging and navigation modality

**Fig. 3:**
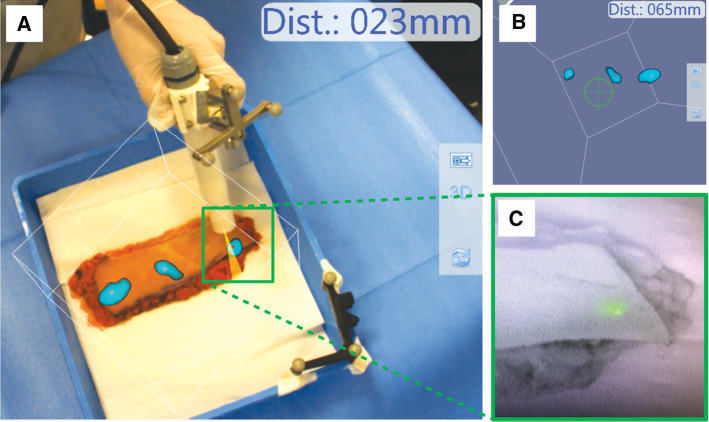
Ex vivo human skin evaluation of the fhMPI modality. **a** Augmented reality overlay of the fhMPI scan on the target anatomy. The calculated distance between the magnetometer probe tip and the centre of the tracer hotspot that is pointed at, is displayed in the upper right corner of the image. **b** Virtual reality navigation towards the tracer hotspots detected in the image, again with the distance towards the hotspots displayed in the upper right corner of the image. **c** Confirmation of the surgical target using fluorescence imaging

Figure 3 visualizes the first feasibility of fhMPI imaging and navigation in human skin tissue. Scan reconstruction was performed ‘on-the-fly’, immediately visualizing and updating the results during scan acquisition. The reconstructed fhMPI scans were visualized in two ways: (1) an augmented reality overlay on the target anatomy, providing the surgeon with insight as to where the SPION-hotspots were found in the tissue (Fig. 3a); (2) a 3D virtual reality view, which could be used to navigate the magnetometer probe towards the individual SPION-hotspots (Fig. 3b), while the real-time read-out of the probe provided audible feedback. Finally, fluorescence imaging confirmed the location of the SPION-hotspots (Fig. 3c).

**Fig. 4:**
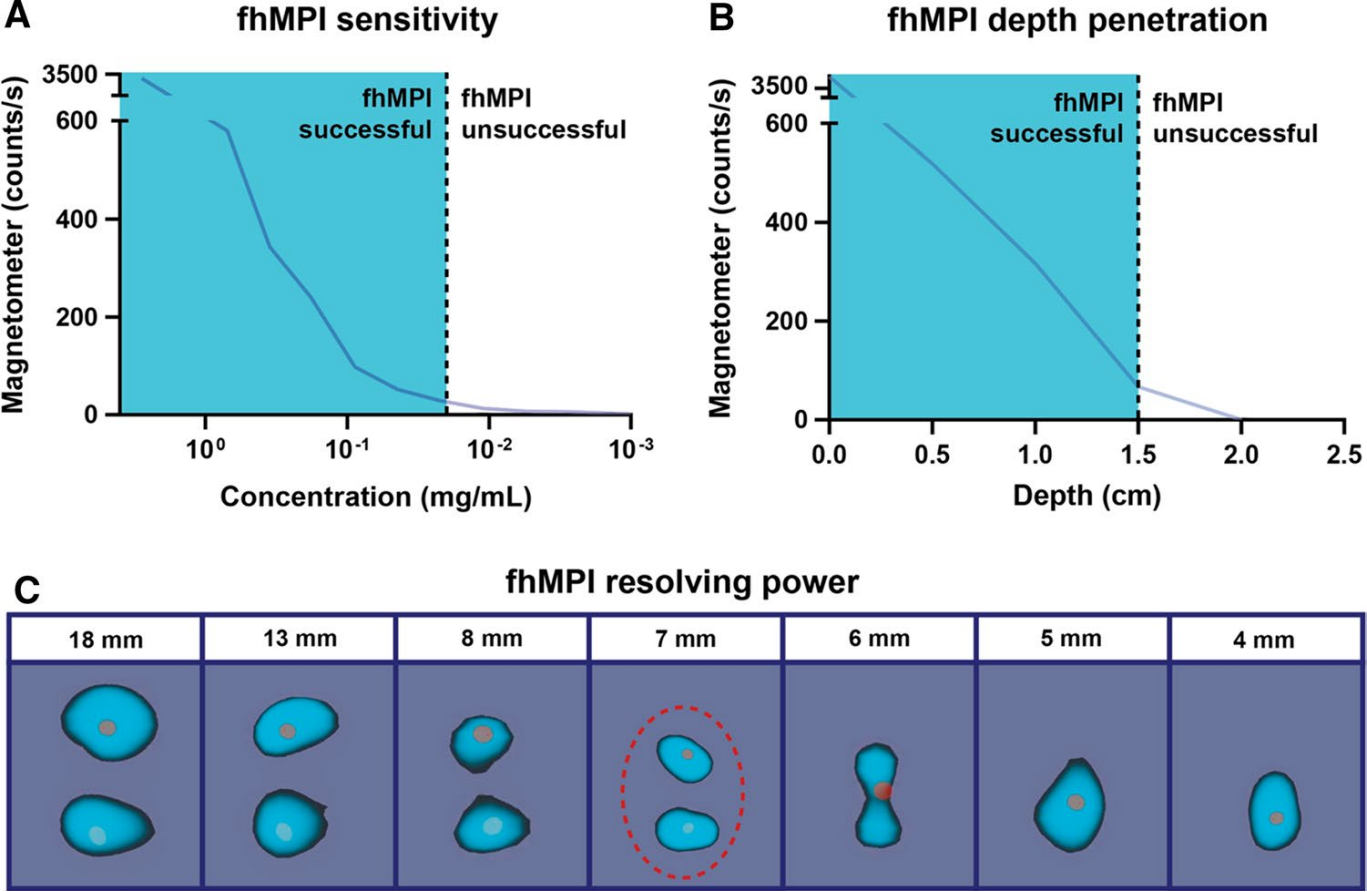
Characterization of the fhMPI modality. **a** fhMPI sensitivity, displaying the minimal SPION concentration needed for successful imaging. **b** fhMPI depth penetration, showing successful scanning is still possible up to 1.5 cm. **c** Overview of the resolving power results, showing that two lymph-node-like targets were still resolvable when the edges of the nodes were at a distance of at least 7 mm

Further evaluation of the fhMPI imaging characteristics is visualized in Fig. 4. The success of fhMPI imaging was strongly related to the sensitivity of the magnetometer probe used, indicating a cut-off at 2.2 × 10^–2^ mg/mL (Fig. 4a). At this range, the magnetometer probe counts dropped below 20. Measurements for the resolving power revealed that two lymph-node-like targets could be resolved as individual targets with an edge-to-edge distance ≥ 7 mm  (Fig. 4c). The fhMPI depth penetration allowed to find tracer hotspots up to roughly 1.5 cm deep (Fig. 4b).

### In vivo porcine evaluation and pathology

**Fig. 5:**
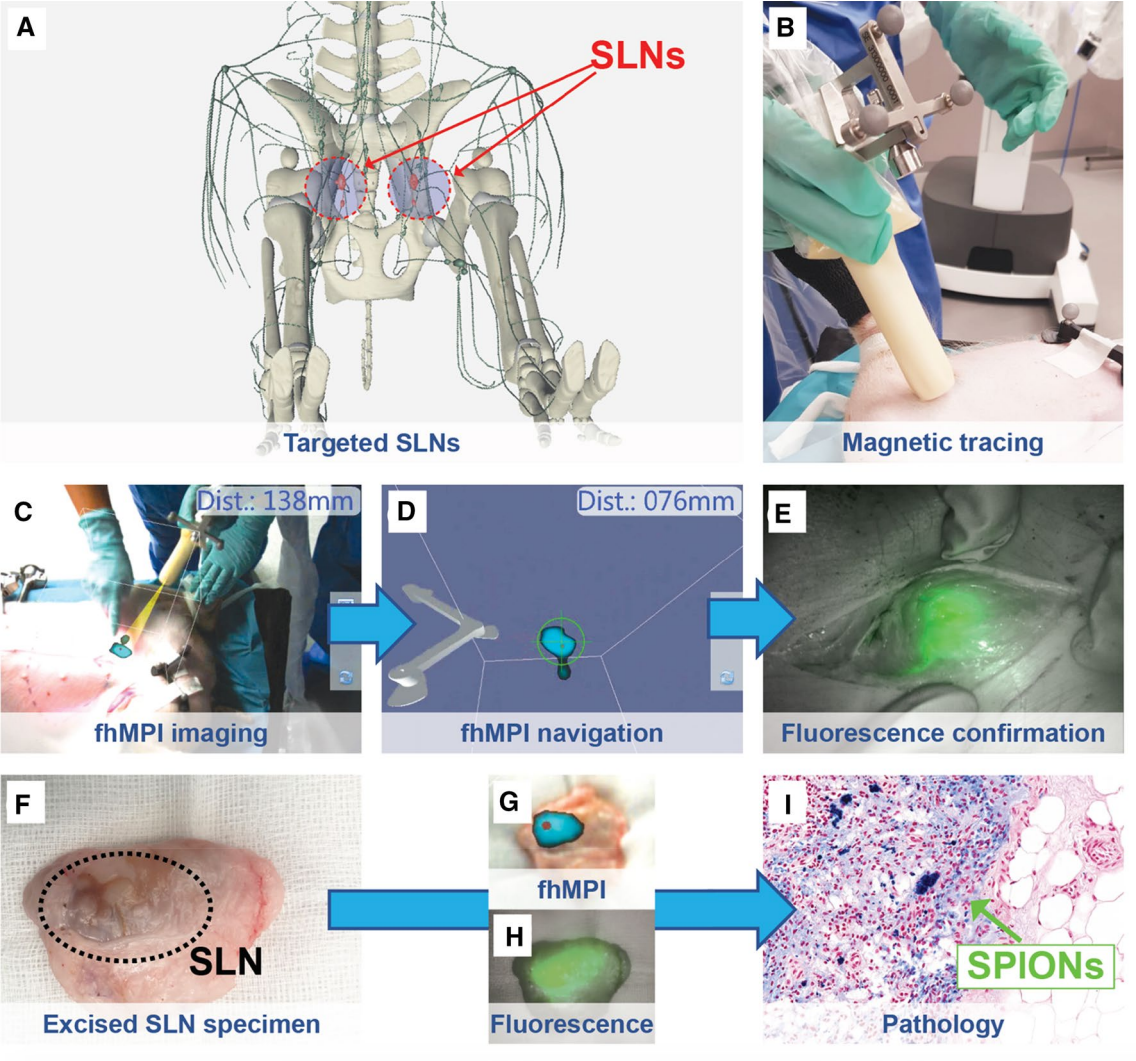
In vivo porcine evaluation of the fhMPI modality. **a** Targeted SLNs displayed within the anatomy. **b** Surgical localization starts with rough magnetic tracing. **c** This is followed with a fhMPI scan in the area of interest, displaying the SLN location registered as an augmented reality overlay on the anatomy. **d** Virtual reality navigation towards the lymph node location. **e** Fluorescence imaging confirms the lymph node location once the tissue is exposed. **f** Ex vivo evaluation depicts a typical SPION-brownish colour in the specimen. **g** SPION uptake confirmed with fhMPI. **h** SPION uptake confirmed with fluorescence. **i** Histopathology confirms the SPIONs (in blue) are indeed in the resected lymph node

Figure 5 shows an overview of the in vivo fhMPI evaluation in the porcine models. In the four groins, a total of five SLNs were localized in the superficial inguinal lymph node groups. There was non-visualization in one groin, something that is in line with clinical SLN procedures [11].

Localization of the SLNs started with probing the unopened groins of the pigs using the magnetometer probe, relying on the probe’s audible feedback. Local signals became stronger when the probe was pressed into the tissue. Once a potential hotspot was identified, a fhMPI scan was acquired (~ 3 min). The ability to visualize a clear hotspot in augmented reality using fhMPI helped boost the surgical confidence with regard to the nodal location (Fig. 5c). Virtual reality navigation based on this fhMPI scan allowed for an estimation of the SLN depths prior to incision, as well as guidance during the incision (Fig. 5d). All the SLNs localized using magnetic tracing were located too deep below the skin surface to allow for direct identification using fluorescence imaging. This changed once the node locations were exposed, which allowed for confirmation with fluorescence imaging (Fig. 5e). Ex vivo examinations revealed that all resected nodes were magnetic and fluorescent. They also all had a (slightly) brownish colouration, which is typical for SPION uptake in the lymph nodes [29]. Histopathologic examination of the specimens additionally confirmed the presence of SPIONs within the lymphatic tissue (Fig. 5i).

## Discussion

In this study, we’ve successfully built and evaluated a novel imaging and navigation modality (i.e. fhMPI) for the intraoperative detection of SPION tracers. This could expand the guidance possibilities during magnetic SLN procedures for various oncological indications (e.g. penile, breast, melanoma, head-and-neck, prostate and vulva cancer). By creating the possibility of hybrid imaging, fhMPI and the related navigation options, intraoperative magnetic imaging is now moving towards the technical advances previously made in radioguided SLN resections.

With the introduction of fhMPI, the family of freehand molecular imaging has been expanded with a fourth modality. As previously reported for fhSPECT [9, 24], freehand beta particle imaging (fhBeta) [30–32] and freehand fluorescence (fhFluo) [14], there are some interesting characteristics that place fhMPI in the context of the other freehand methods. First, image resolving power is highest with fhFluo with respect to fhSPECT and fhMPI (1 mm, 6 mm and 7 mm, respectively) [14]. The image resolving power for fhBeta is not reported, but expected to be around 1 mm [8]. Second, depth penetration of fhMPI and fhSPECT is higher than with fhFluo (1.5 cm, > 1.5 cm and 0.5 cm, respectively) [14]. The depth penetration of fhBeta is not reported, but expected to be only a few mm’s [8]. Based on these parameters, fhMPI makes an interesting clinical addition to the available options.

All SLNs found in the in vivo porcine models were both magnetic and fluorescent. Hence, this feature opens up the possibility to see if a hybrid concept involving MRI, magnetic tracing and fluorescence imaging could provide an alternative to those using fluorescence imaging and radioguided surgery [15].

Limiting in this study was the lack of porcine preoperative imaging, making it possible that not all stained SLNs were localized during surgery. When the concept is evaluated in clinical routine, however, preoperative SLN mapping can be realized using MRI, providing a roadmap for surgery, something that has proven critical in the radioactive SLN approach [13].

## Conclusions

Freehand magnetic particle imaging is feasible in vivo, providing a 3D imaging and navigation modality for magnetic nanoparticles in the operating room. In addition, our findings show that the additional use of ICG provides the ability to refine the procedure via integrated fluorescence imaging. Further clinical evaluation should confirm this also provides added benefit in human patients.

## Data Availability

Data are available on reasonable request.
